# Cs02526 effector: A double-edged sword in mulberry–pathogen interactions

**DOI:** 10.1093/plphys/kiae309

**Published:** 2024-05-29

**Authors:** Ritu Singh

**Affiliations:** Assistant Features Editor, Plant Physiology, American Society of Plant Biologists; Department of Plant Science, University of California, Davis, CA 95616, USA

For millions of years, there has been a subtle yet intense competition between pathogens and plants. Phytopathogens proliferate by usurping nutrients from plant tissues while host plants deploy diverse defense mechanisms to impede pathogen proliferation. Plants defend themselves using pattern recognition receptors (PRRs) to recognize pathogen-associated molecular patterns (PAMP), initiating PAMP-triggered immunity (PTI) to restrict pathogen growth. As a counter-response, phytopathogens translocate effector proteins into plant cells to sabotage the host cellular machinery and promote infection (Khan et al. 2018). However, these effectors can be recognized by host cytosolic NOD-like receptors, activating effector-triggered immunity (Jones and Dangl 2006; Cui et al. 2015). Extensive studies have highlighted the crucial role of phytopathogen effectors in plant infestation, as these effectors target various host macromolecules and manipulate them through diverse enzymatic activities. Thus, understanding the effector function is crucial for developing novel strategies to manage plant diseases.

Mulberry (*Morus* spp.) trees are economically important, particularly in the silkworm (sericulture) industry, and their fruits are rich in nutrients and anthocyanins (Li et al. 2020). However, mulberry sclerotinia disease, primarily caused by the necrotrophic fungus *Ciboria shiraiana*, poses a significant threat, leading to substantial annual losses in mulberry fruit yield (Zhu et al. 2021; Zhang et al. 2021a, 2021b). In the current issue of *Plant Physiology*, Zhang et al. (2024) shed light on potential solutions by identifying key effectors in *C. shiraiana* and investigating their impact on plant immune responses.

The authors initially identified potential effectors in *C. shiraiana* by analyzing its genome followed by transiently expressing some of these effectors in *Nicotiana benthamiana* plants. One of these effectors, *Cs02526*, induced cell death in *N. benthamiana*. To further validate this cell death response, Zhang and coworkers assessed electrolyte leakage and the expression levels of hypersensitive response (HR)-specific marker genes in *N. benthamiana* leaves agroinfiltrated with the *Cs02526* construct. The findings revealed a significant increase in electrolyte leakage and the expression of the HR-marker genes in the *Cs02526* agroinfiltrated leaves compared with the control, indicating that *Cs02526* can induce cell death in *N. benthamiana*. Further, investigations demonstrated that *Cs02526* could also induce cell death in various plant species, including tobacco, tomato, strawberry, and mulberry leaves, suggesting its broad activity across different plants.

Additionally, expression of the gene encoding Cs02526 increased during infection, suggesting its role in pathogenicity. To confirm its role in pathogenicity, authors generated *Cs02526*-silenced strains in *C. shiraiana* through an RNAi-mediated approach. The inoculation of detached leaves with mycelial plugs revealed reduced lesions and pathogen biomass in *Cs02526*-silenced strains compared with wild type, confirming that *Cs02526* is required for pathogenicity in *C. shiraiana*.

*Cs02526* encodes a protein of 86 amino acids, including an N-terminal signal peptide (SP). To understand the mode of action of *Cs02526* and determine the significance of the SP, the authors agroinfiltrated *Cs02526* without signal peptide (*Cs02526ΔSP*) into *N. benthamiana* leaves. Compared with *Cs02526*, *Cs02526ΔSP* failed to trigger cell death, indicating the crucial role of SP for *Cs02526*-induced cell death. Further, subcellular localization indicated that Cs02526 is an apoplastic effector, whereas Cs02526ΔSP failed to localize in the apoplast, suggesting that Cs02526 must be targeted to the extracellular space of plant tissue to induce cell death.

Given Cs02526 localization in the plant apoplast, Zhang and coworkers speculated that it probably functions akin to PAMPs. To verify this assumption, the expression levels of PTI and defense marker genes were determined using qRT-PCR, revealing a significant increase in PTI marker genes. Furthermore, the expression of defense marker genes associated with plant hormone signaling pathways, particularly salicylic acid (SA), jasmonic acid (JA), and ethylene (ET)-dependent immunity, were significantly upregulated in *N. benthamiana* and mulberry leaves agroinfiltrated with *Cs02526*. These results indicated that *Cs02526*- induced PTI in plants and activated defense pathways mediated by SA, JA, and ET. To further determine if *Cs02526* induces cell death in plants lacking canonical PRRs, the authors generated virus-induced gene silencing (VIGS) lines targeting *BAK1* and *SOBIR1* PRRs. They found that *Cs02526*-induced cell death was abolished in the *BAK1*-silenced lines, while it remained unaffected in the *SOBIR1*-silenced and control lines. This suggests that *BAK1* is essential for *Cs02526*-triggered immune responses and also supports the model that *Cs02526* behaves like a PAMP.

The authors also explored the potential of Cs02526 as a virulence factor in biological control using the spray-induced gene silencing method. Initially, the efficiency of dsRNA uptake in *C. shiraiana* was confirmed, followed by treatment with dsRNA-*Cs02526*, resulting in significantly smaller lesions in *N. benthamiana* and mulberry leaves compared with the control. The results indicated Cs02526 as a potential new RNAi target for controlling plant diseases. Moreover, the researchers found that a low concentration of Cs02526 could enhance plant disease resistance, as treatment of *N. benthamiana* leaves with recombinant Cs02526 protein led to significantly reduced lesion areas and pathogen biomass compared with the control. Additionally, Cs02526-treated *N. benthamiana* plants also showed resistance to *Sclerotinia sclerotiorum* compared with untreated. These findings collectively suggested that Cs02526 can enhance plant resistance to various pathogens, especially from the Sclerotiniaceae family.

In conclusion, this study highlighted that *C. shiraiana* secretes the apoplastic effector *Cs02526*, which not only induces robust immune responses in plants (cell death) but also enhances pathogenicity, facilitating *C. shiraiana* infection. Spraying of dsRNA-*Cs02526* reduces the transcript level of *Cs02526*, contributing to weak pathogenicity. Conversely, treating plants with exogenous low concentrations of Cs02526 protein induces plant immunity and disease resistance (Fig. 1). Cs02526 emerges as a promising target for controlling plant diseases and holds potential for the development of innovative biological fungicides. However, further research is needed to better understand the optimal concentrations required for effective disease management.

**Figure 1.**
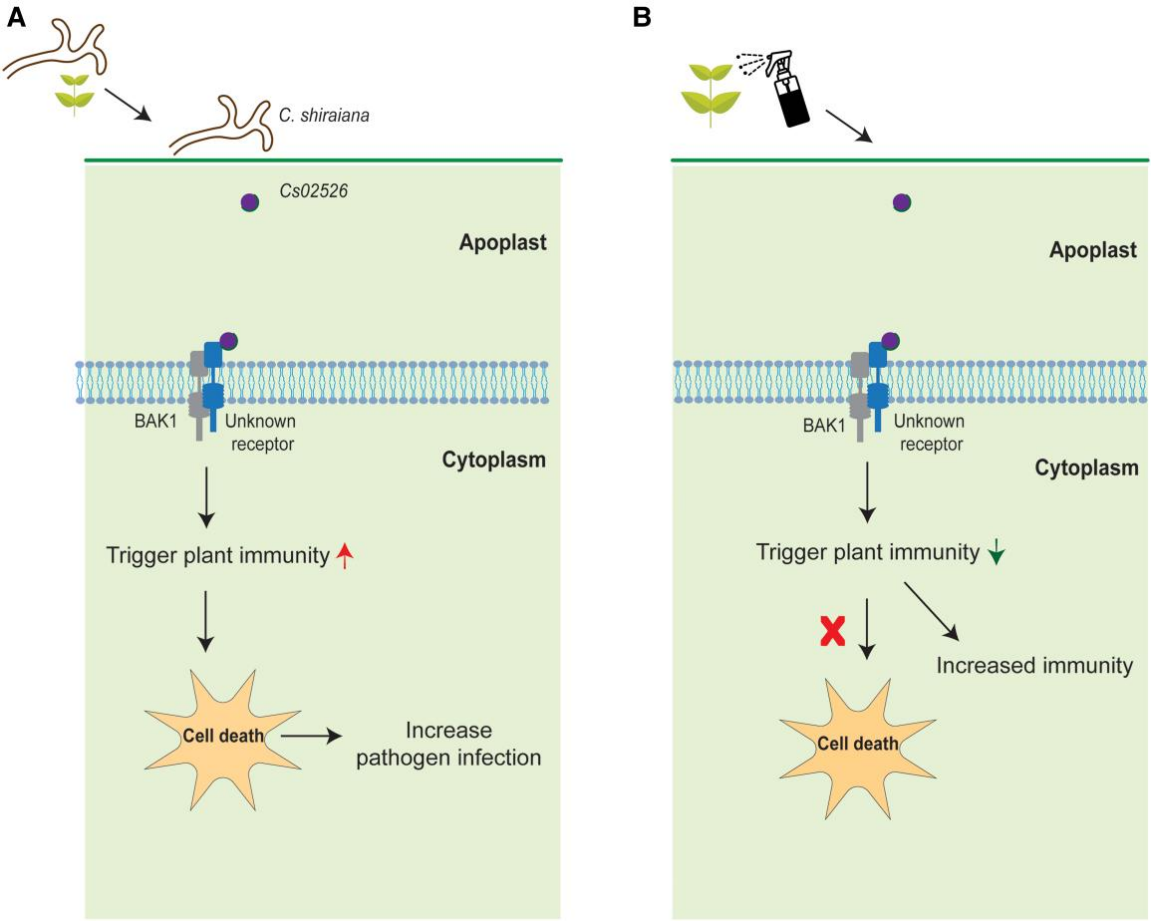
Schematic representation of *Cs02526* function in *C. shiraiana* infection and its role in plant immunity (adapted from Figure 7 of Zhang et al., 2024). **A)***Cs02526*, an apoplastic effector from *C. shiraiana*, is secreted and recognized by PRR receptors, triggering intense plant immunity and cell death, thus facilitating pathogen infection. **B)** Application of a low concentration of exogenous Cs02526 protein stimulates a mild plant immune response, without inducing cell death, thereby enhancing resistance against pathogens.

## Data Availability

No data is generated in this study.
